# The Disadvantage of High-Tension Circuits for Dental Work, and How They May Be Altered to Circuits of Low Potential, Suitable for Dental Operations

**Published:** 1893-07

**Authors:** Peter Brown

**Affiliations:** Montreal, Canada


### THE DISADVANTAGE OF HIGH-TENSION CIRCUITS FOR DENTAL WORK, AND HOW THEY MAY BE ALTERED TO CIRCUITS OF LOW POTENTIAL, SUITABLE FOR DENTAL OPERATIONS.

BY DR. PETER BROWN, MONTREAL, CANADA.

THE tendency of central stations to increase the potential of their power-circuits raises a new complication in the application of electricity to dental practice. In the first place, it increases the dangers of the operator and patient getting a shock from accidental contact with exposed parts of the apparatus; then it necessitates the putting of lamps or resistance-coils in the circuit, in order to reduce it low enough to render it safe to use about the mouth; this involves additional expense for rheostats and resistance-lamps, and complicates the wiring.

The usual voltage of power-circuits is now from two hundred and twenty to five hundred volts; it depends in some cases on the distance to which the line is carried from the central station, and in others on the use to which the circuit is put. For stationary motors the two-hundred-and-twenty-volt circuit is usually used, and street-railways use the five-hundred-volt circuit; either of these may be economically transformed into low potential circuits, which can be safely used in all operations where electricity is indicated, or where it is desirable to use it for motive-power.

The method used by the writer is as follows: A half horse-power motor, running off a two-hundred-and-fifty-volt circuit, is belted to a counter-shaft; this shaft runs a quarter horse-power, one-hundred-and-ten-volt dynamo, and a sixth horse-power, ten-volt dynamo. In the first dynamo we get a current of two and a half ampères at one hundred and ten volts potential, and in the second a current of ten ampères at ten volts potential. Two sets of wires are led from this miniature central station, which is placed in the basement of the office, to the chair where the one-hundred-and-ten-volt current is used to run a dental engine motor, a lathe motor, and a fan which is used on warm days to keep the air in circulation. The current from the ten-volt generator is used to run the electric mallet, mouth-lamp, root-dryer, cautery, and gold annealer. By this arrangement the two ampères of current which enters the office at a potential of two hundred and fifty volts is transformed and divided into a current of ten ampères at ten volts potential, and a current of two and a half ampères at one hundred and ten volts. Without this means of transforming the power it would be a difficult matter to use such a high-tension circuit as two hundred and fifty volts to heat a cautery or to use it for a root-dryer or mallet. Where one has to pay for his electric power by meter, it would cost a great deal to use it for cautery purposes, as the heating power of a current depends on its quantity or number of ampères, and not on its potential or number of volts, and it is the ampères we pay for. To use a current of two hundred and fifty volts, we would have to put five fifty-volt—sixteen-candle-power—lamps in series, then we would get a current of one ampère, and the voltage of the current would be very much reduced; but this involves a great waste of energy, as we are expending the greatest part of the current in producing light which is not wanted at the time, and we have the additional expense of maintaining lamps. If we want a greater current than one ampère, we remove the sixteen-candle-power lamps and put in thirty-two-candle-power lamps instead; this will give us two ampères; and we can go on increasing the quantity indefinitely until the maximum capacity of the circuit is reached by using several banks of lamps. This is one method of reducing it, and it is usually the most satisfactory. The fifty-two-volt alternating circuit may be adapted for cautery purposes by a small converter; a current of two ampères, at fifty-two volts, can be transformed into a current of ten ampères and five volts.

There is now on the market a machine called a motor-dynamo or direct-current transformer, made by the Crocker-Wheeler Electric Company of New York. It consists of a motor with a double-wound armature; one side is made to receive a high-pressure current, the other to give it out at a low pressure. The idea is the same as mentioned in the beginning of this article, only that the motor and dynamo are on one shaft instead of being connected by means of counter-shaft and belt. A machine of this kind would be the proper thing for our purpose, as we could have one made to receive any current which might be convenient for us to work from, and give it out at any pressure we might decide on, say ten volts. We could then contract for one ampère of current at two hundred and fifty volts, or half an ampère at five hundred volts, or two and a half ampères at one hundred and ten volts. Either of these would give us a quarter horse-power; the dynamo side of the machine would then give us twenty-five ampères at ten volts, or two hundred and fifty watts; and this is just what we want,—as was mentioned before, a current of low pressure and large quantity. This machine can be placed in an adjoining room, or on a shelf in the basement, or in any convenient place. As it is almost noiseless in its action, it could be placed in the office, provided that it could not be interfered with by curious people, although their curiosity might receive a check if they got hold of the primary or high-pressure side of the apparatus. Once the apparatus is installed, we have nothing to do but turn the handle of the starting-box and leave it alone until we are through for the day. It will take care of itself. The wires from the dynamo side are brought to the chair and connected with our apparatus; for the engine we can have a one-eighth-horse-power ten-volt motor with its regulating-box or rheostat; this will require about ten ampères to run it, according to the work we give it to do; then we have still fifteen ampères to draw on for any other purpose we may wish to use it for, and we are absolutely safe from shock.

Another method of transforming a current is to use storage batteries. We can connect the battery to the power-line and put in suitable resistance, and take our power from the battery as we require it. This is not so satisfactory as using a motor-generator, but it is mentioned so that one can use it if he wishes.

If one is so situated that he cannot get power from a direct current, but can get the alternating current, it may be used by putting in an alternating-current motor of a half horse-power and belting it to a direct-current dynamo wound for any voltage we may desire to use; fifteen volts should be about the maximum voltage used about the dental chair. Alternating-current motors of one horse-power are now on the market. A motor of this class of about one-half horse-power would be ample for all the requirements of dental work. So far small motors for the alternating current have not been a success, and it cannot be used for the electric mallet at all, no matter how we reduce it; but it may be used very satisfactorily for cautery work by having a small converter made by which we could change a current of fifty two volts and two ampères into one of ten ampères and ten volts; the number of watts remains about the same,—that is, one hundred, allowing a loss of four per cent. in the converter.

